# Channel Defect Profiling and Passivation for ZnO Thin-Film Transistors

**DOI:** 10.3390/nano10061186

**Published:** 2020-06-18

**Authors:** Soo Cheol Kang, So Young Kim, Sang Kyung Lee, Kiyung Kim, Billal Allouche, Hyeon Jun Hwang, Byoung Hun Lee

**Affiliations:** 1 Center for Emerging Electronic Devices and Systems (CEEDS), Gwangju 61005, Korea; kangsc817@etri.re.kr (S.C.K.); una0918@gist.ac.kr (S.Y.K.); leesk@gist.ac.kr (S.K.L.); kky0511@gist.ac.kr (K.K.); allouche_bilal@hotmail.fr (B.A.); hhjune@gist.ac.kr (H.J.H.); 2 School of Materials and Science Engineering, Gwangju Institute of Science and Technology (GIST), Gwangju 61005, Korea; 3 DMC Convergence Research Department, Electronics and Telecommunications Research Institute (ETRI), Daejeon 34129, Korea

**Keywords:** ZnO thin-film transistors, oxygen vacancy, vacuum treatment, oxygen annealing, trap-induced Schottky barrier lowering

## Abstract

The electrical characteristics of Zinc oxide (ZnO) thin-film transistors are analyzed to apprehend the effects of oxygen vacancies after vacuum treatment. The energy level of the oxygen vacancies was found to be located near the conduction band of ZnO, which contributed to the increase in drain current (I_D_) via trap-assisted tunneling when the gate voltage (V_G_) is lower than the specific voltage associated with the trap level. The oxygen vacancies were successfully passivated after the annealing of ZnO in oxygen ambient. We determined that the trap-induced Schottky barrier lowering reduced a drain barrier when the drain was subjected to negative bias stress. Consequentially, the field effect mobility increased from 8.5 m^2^ V^−1^·s^−1^ to 8.9 m^2^ V^−1^·s^−1^ and on-current increased by ~13%.

## 1. Introduction

Zinc oxide (ZnO) is an amorphous oxide semiconductor that can be economically patterned via simple wet chemical etching at low temperatures. ZnO has several favorable properties such as nontoxicity, flexibility and transparency [1,2,3,4]. Owing to these benefits, ZnO was extensively studied for applications in various thin-film devices such as nanogenerators, thermal and pressure sensors, flexible devices, memory devices and logical circuits.

In particular, ZnO was investigated as a promising alternative to the polysilicon channel materials used in backplane thin-film transistors (TFTs) because of its high mobility ~20 cm^2^ V^−1^∙s^−1^ [5,6]. Various composite materials containing ZnO such as InGaZnO and InSnZnO have also been studied for this application [7,8,9]. In general, the electrical characteristics of TFTs, having an amorphous oxide semiconductor channel including ZnO, show a strong ambient dependence during the fabrication process [10] and electrical operation [11,12], which were attributed to the various oxygen vacancies such as V_O_, V_O_^+^ and V_O_^2+^. These oxygen vacancies can exist at different energy levels: V_O_ at 0.4 eV from the valence band, V_O_^+^ at 2.56 eV from the valence band and V_O_^2+^ at 3.2 eV from the valence band [13,14]. These oxygen vacancies serve as active trap sites and cause various reliability problems [15,16,17]. Although several countermeasures for this problem have been developed using passivation layers such as SiO_2_ and HfO_2_ [18,19], the detailed mechanisms of device degradation and recovery related with the oxygen vacancies have not been systematically investigated.

In this study, the non-passivated ZnO TFTs were fabricated using atomic layer deposition (ALD) and the influences of various oxygen treatments and field stress were investigated to determine the correlation between the electrical characteristics and the oxygen vacancies. The energy level of reversible oxygen vacancies in ZnO was found to be near the conduction band. Furthermore, we observed that the field–effect mobility and on-current were increased by a trap-induced Schottky barrier lowering after the drain was subjected to negative bias stress [20].

## 2. Materials and Methods

**Figure 1:**
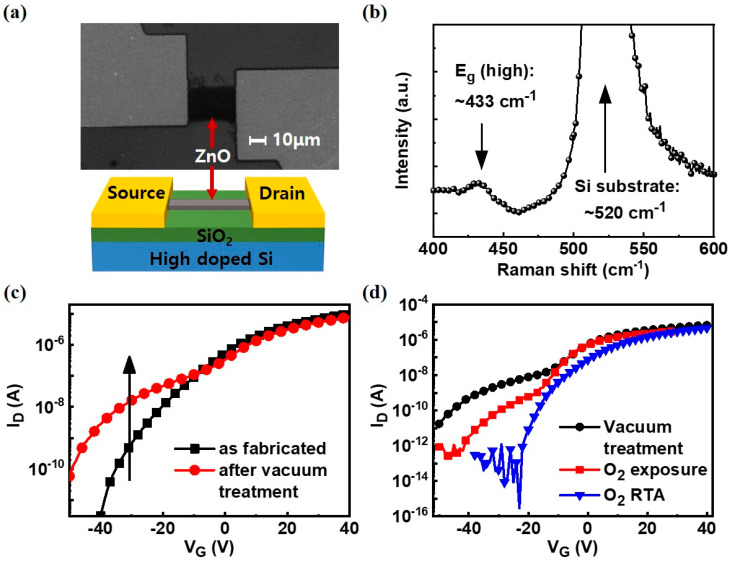
(**a**) SEM image of back gate ZnO thin-film transistors (TFT) and the schematic of the device structure. Channel width and length are 25 μm and 30 μm, respectively; (**b**) Raman spectrum of ZnO channel showing ZnO related peak at ~433 cm^−1^; (**c**) representative transfer curves before (black) and after (red) the vacuum treatment at ~10^−3^ Torr for 24 h; (**d**) transfer curves after different treatments: Black circle line denotes the effect of low vacuum treatment; the red square, the sample exposed to oxygen atmosphere at 1 bar pressure and room temperature (25 °C) for 24 h; blue triangle, the effect of a rapid thermal annealing (RTA) system at 150 °C for 30 min, where the oxygen flow was maintained at 500 sccm.

For back gate device fabrication, SiO_2_ (90 nm)/highly p-doped silicon substrate (resistivity < 0.005 Ω cm) was first cleaned in SC1 solution (NH_4_OH:H_2_O_2_:H_2_O = 1:1:5 at 80 °C for 10 min). Then, 50-nm-thick ZnO was deposited using ALD with 300 cycles of DEZ (0.2 s)/N_2_ purge (10 s)/H_2_O (0.6 s)/N_2_ purge (10 s) at 120 °C. ZnO channel was then patterned using photolithography and diluted HCl etchant. Finally, Au source and drain electrodes were deposited by an e-beam evaporator that was 100-nm-thick and patterned via photolithography and metal wet etching process. The SEM image of ZnO TFTs and the schematic of the device structure are shown in Figure 1a. The quality of ZnO was verified by Raman spectroscopy (514 nm) and a typical Raman peak of ZnO was observed at ~433 cm^−1^ [21] as shown in Figure 1b.

In order to analyze the effect of oxygen vacancies, three different treatments were used. First, three samples were stored in a vacuum chamber (~10^−3^ Torr, 24 h) to intentionally create oxygen vacancies. One of them was then exposed to oxygen for 24 h (1 bar, 30 °C) to reduce the oxygen vacancies. The next sample was annealed in oxygen atmosphere using rapid thermal annealing (RTA) process (30 min, 150 °C, 500 sccm) to accelerate the reduction of oxygen vacancies. The annealing temperature, 150 °C, was deliberately selected to be slightly above the deposition temperature to make this process compatible with flexible electronics (<200 °C).

The electrical characteristics were measured at 30 °C in ambient air. For detailed device characterization, the following assessments were performed: (i) Reliability assessments to determine the effect of the increased oxygen vacancies after vacuum treatment on the Schottky barrier; (ii) The ZnO TFTs stored in vacuum were subjected to a negative drain bias stress, to investigate the degradation of the Schottky barrier (V_D.stress_ = −2.5 V, V_G.stress_ = 0 V); (iii) The effect of stress on the channel/drain junction was investigated by measuring the I_D_–V_G_ transfer curve and the capacitance between the source and the drain.

The I_D_–V_G_ measurement and stress application were performed using stress and measurement method of Keithley 4200-SCS parameter analyzer and the delay between the stress and measurement was less than a few tens of a millisecond. Capacitance–voltage curves were measured using an Agilent 4294A impedance analyzer.

## 3. Results and Discussions

ZnO TFTs with an Au electrode showed n-type device characteristics as shown in Figure 1c. The field-effect mobility extracted from the as fabricated device shown in Figure 1c was approximately 10 cm^2^/V∙s, which is similar to the values reported in the literature [22,23]. Field-effect mobility was calculated using μ_FE_ = (L/W ∙ C_ox_ ∙ V_D_) (∂I_D_/∂V_G_). After the vacuum treatment, a “hump” (red line) was observed at the low field region of I_D_ and the I_off_ increased by three orders of magnitude from 3.1 × 10^−12^ A to 2.8 × 10^−9^ A at V_G_ = −40 V. When the ZnO TFTs were exposed to the oxygen ambient at 1 bar for 24 h at 30 °C, the hump was reduced primarily at the subthreshold region as shown in Figure 1d. When the ZnO TFTs were annealed at 150 °C for 10 min in oxygen ambient, the hump at the subthreshold region almost disappeared, indicating that the hump at the subthreshold region is due to the oxygen vacancies, which can be reversibly controlled by oxygen treatment. Moreover, the electrical properties of the ZnO TFTs (I_on_/I_off_, V_T_, Subthreshold Slope) were deteriorated after the vacuum treatment and improved again after O_2_ exposure or O_2_ RTA as shown in Table 1.

**Figure 2:**
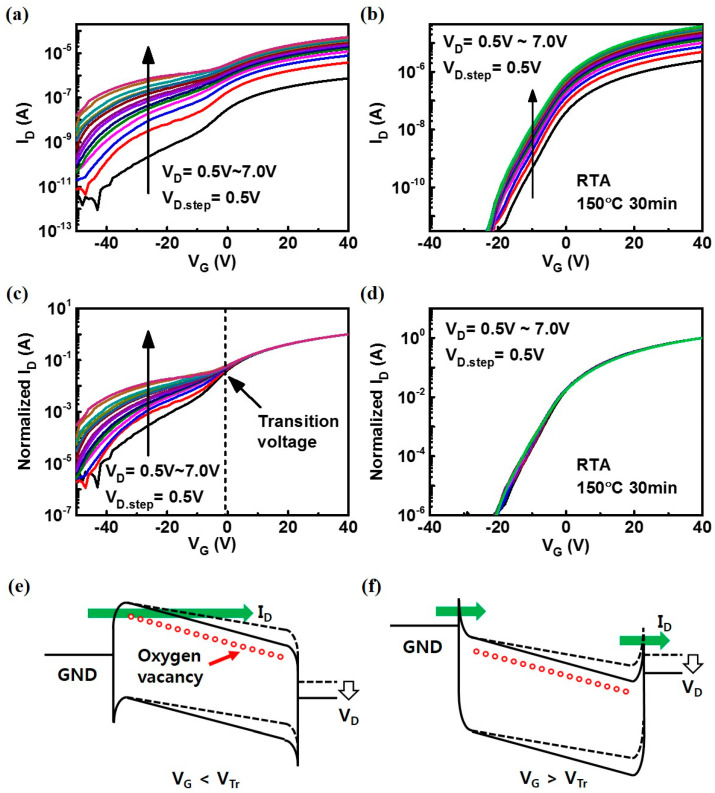
I_D_–V_G_ transfer curves measured at different V_D_ (**a**) after low vacuum treatment (**b**) after RTA in oxygen ambient (150 °C for 30 min); (**c**,**d**) I_D_–V_G_ transfer curves of (**a**) and (**b**), respectively, normalized with respect to I_D_ at V_G_ = 40 V; (**e**) Schematic band diagram for the case having V_G_ below the transition voltage (V_Tr_). Trap-assisted tunneling (TAT) current (green arrow) increased via an oxygen vacancy level near the conduction band of ZnO; (**f**) Schematic band diagram for the case having V_G_ above V_Tr_. The current (green arrow) flows through the Schottky barrier at source/channel and channel/drain without being affected by oxygen vacancies. The small red circles denote the expected energy level of oxygen vacancy.

After identifying the various oxygen treatment methods to obtain devices having different levels of oxygen vacancies, their electrical characteristics were investigated in detail. First, the transfer curves (I_D_–V_G_) of devices stored in vacuum and annealed in oxygen ambient (annealed at 150 °C for 30 min) were further examined as shown in Figure 2a,b, respectively. Figure 2a shows the gradual I_D_ increase as a function of V_D_, which shows a gradual saturation and much significant increase at the low gate bias region. After the oxygen annealing, this device showed weaker V_D_ dependence compared with the device stored in a vacuum.

The difference between the two groups becomes more evident with the normalized I_D_–V_G_ curves as shown in Figure 2c,d. When the drain current curves measured at various V_D_ are normalized with maximum Id value at the V_G_ = 40 V, all I–V curves overlap each other. This result indicates that the relative difference in the drain current was due to the difference in the effective drain bias, i.e., series resistance component. In contrast, in a low field region, apparent drain current increase as a function of V_D_ was observed in the devices stored in vacuum, which implies that the oxygen vacancies primarily contribute to the drain current increase at the low field region. These results also confirm that the oxygen vacancies generate shallow trap sites that contribute to the trap-assisted tunneling (TAT) from the source to the channel as schematically shown in Figure 2e [24,25]. At a high field region, these shallow traps do not directly contribute to the drain current because the carrier tunnel through the Schottky barrier becomes dominant as shown in Figure 2f. This model explains the gate bias dependence of oxygen vacancy induced hump and why they do not appear at the gate bias region above the specific bias marked in Figure 2c as a critical voltage, V_Tr_. These results match those of the previous study on the impact of oxygen vacancy in ZnO particles [26,27].

**Figure 3:**
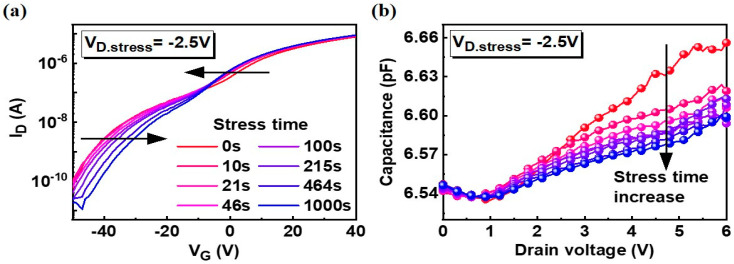
(**a**) I_D_–V_G_ transfer curves after negative bias stress. V_D.stress_ = −2.5 V and stress time = 1000 s. Curves were shifted from the red to the blue line as indicated by the arrows; (**b**) gradual change in the source to drain capacitance as a function of drain voltage during the constant voltage stress. Capacitance was measured at V_G_ = 0 V.

**Figure 4:**
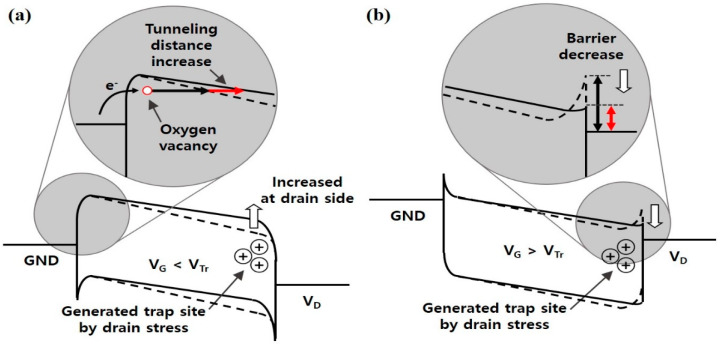
(**a**) Energy band diagram when the V_G_ is lower than the V_Tr_ with the enlarged region at the source/channel side. The tunneling distance of TAT (red arrow) increases due to the change in the potential barrier by positive charge traps near the channel/drain side; (**b**) Schottky barrier is lowered due to the charge traps when the V_G_ is larger than the V_Tr_ near the channel/drain side after stress.

Next, to estimate the effects of oxygen vacancies on the Schottky barrier at the channel/drain junction, constant voltage stress was performed as shown in Figure 3. In this process, negative drain bias stress was applied to the drain side (V_D.stress_ = −2.5 V, V_G.stress_ = 0 V) to probe the degradations in the channel/drain junction region. As the stress time increased, I_D_ gradually changed from the red to the blue line as shown in Figure 3a. The I_D_ drastically decreased at the V_G_ region below V_Tr_ and this trend is reversed at the V_G_ higher than V_Tr_. This drastic decrease can be explained by the migrated positive charges at the drain side as illustrated in Figure 4a, which increases the TAT distance at the source side.

As shown in Figure 3b, the source to drain capacitance decreased from the red to the blue line as the stress time increased. At a higher V_D_, the decrease was more pronounced and the capacitance was decreased from 6.66 pF to 6.60 pF at V_D_ = 6 V. Although the change in the junction capacitance is relatively small, it is apparent that the barrier profile of Schottky junction is indeed affected by the charge trapping as reported in the prior works on trap-induced Schottky barrier lowering [28,29] and illustrated in Figure 4b. Even though we cannot identify the band profile accurately from the capacitance measurement, the drain bias dependence and stress dependence indicates that the band profile shown in Figure 4 matches with the experimental results because the smaller capacitance means the smaller band bending by charge trapping.

**Figure 5:**
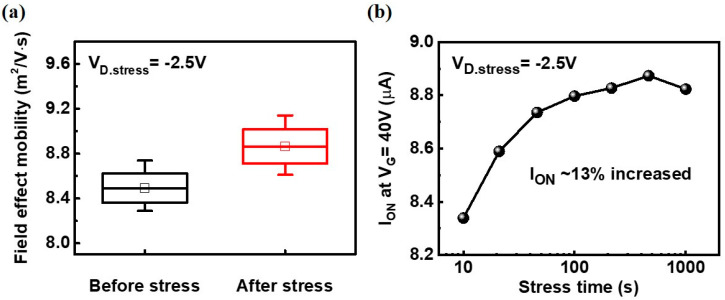
Increase of the field-effect mobility (**a**) and on-current (**b**) after negative drain bias stress with reducing “hump” phenomenon. The field-effect mobility increased from ~8.5 cm^2^ V^−1^∙s^−1^ to ~8.9 cm^2^ V^−1^∙s^−1^ and on-current was increased by approximately 13% after negative drain bias stress by the trap-induced Schottky barrier lowering.

Finally, the impact of constant voltage stress on the field effect mobility and on–current is investigated as shown in Figure 5. Interestingly, the field effect mobility increased after the stress, from 8.5 m^2^ V^−1^·s^−1^ to 8.9 m^2^ V^−1^·s^−1^, because the swing of I_D_–V_G_ curve actually improved as shown in Figure 3a. This result is due to the suppression of subthreshold leakage current caused by the tunneling distance increase at the low gate bias region which can be explained with the band diagram shown in Figure 4a, which in turn improves the swing. The on-current values in the high field region were also enhanced after the stress by ~13% as shown in Figure 5b. This result can be explained by the band diagram shown in Figure 4b. After the negative drain bias stress, the traps accumulated in the drain side improve the channel conductivity due to the trap-induced Schottky barrier lowering effect.

## 4. Conclusions

Various impacts of oxygen vacancies in ZnO channel were investigated. While we confirmed that the oxygen vacancies can be passivated with oxygen anneal, a new understanding of the device operating mechanism was accomplished through constant voltage stress on a drain. We found that the charge trapping in the drain side of the ZnO channel resulted in improved mobility, drain current and swing by modulating the Schottky barrier height and tunneling distance.

## Figures and Tables

**Table 1:** Electrical properties of the ZnO TFT with different condition.

Properties	As Fabricated	Vacuum Treatment	O_2_ Exposure	O_2_ RTA
I_on_/I_off_	3.1 × 10^6^	2.8 × 10^3^	9.2 × 10^5^	1.5 × 10^7^
V_T_ (V)	−9.6	−11.1	−9.8	−4.1
SS (V/dec.)	6.3	10.4	7.5	2.8
